# Tumor Treating Fields (TTFields) Concomitant with Immune Checkpoint Inhibitors Are Therapeutically Effective in Non-Small Cell Lung Cancer (NSCLC) In Vivo Model

**DOI:** 10.3390/ijms232214073

**Published:** 2022-11-15

**Authors:** Yiftah Barsheshet, Tali Voloshin, Boris Brant, Gadi Cohen, Lilach Koren, Roni Blatt, Shay Cahal, Tharwat Haj Khalil, Efrat Zemer Tov, Rom Paz, Anat Klein-Goldberg, Catherine Tempel-Brami, Sara Jacobovitch, Alexandra Volodin, Tal Kan, Bella Koltun, Cfir David, Adi Haber, Moshe Giladi, Uri Weinberg, Yoram Palti

**Affiliations:** Novocure Ltd., Topaz Building, MATAM Center, Haifa 31905, Israel

**Keywords:** Tumor Treating Fields (TTFields), immunogenic cell death, anti-PD-1 therapy, anti-PD-L1 therapy, anti-CTLA-4 therapy, non-small cell lung cancer (NSCLC)

## Abstract

Tumor Treating Fields (TTFields) are electric fields that exert physical forces to disrupt cellular processes critical for cancer cell viability and tumor progression. TTFields induce anti-mitotic effects through the disruption of the mitotic spindle and abnormal chromosome segregation, which trigger several forms of cell death, including immunogenic cell death (ICD). The efficacy of TTFields concomitant with anti-programmed death-1 (anti-PD-1) treatment was previously shown in vivo and is currently under clinical investigation. Here, the potential of TTFields concomitant with anti- PD-1/anti-cytotoxic T-lymphocyte-associated protein 4 (anti-CTLA-4) or anti-programmed death-ligand 1 (anti-PD-L1) immune checkpoint inhibitors (ICI) to improve therapeutic efficacy was examined in lung tumor-bearing mice. Increased circulating levels of high mobility group box 1 protein (HMGB1) and elevated intratumoral levels of phosphorylated eukaryotic translation initiation factor 2α (p-eIF2α) were found in the TTFields-treated mice, indicative of ICD induction. The concomitant application of TTFields and ICI led to a significant decrease in tumor volume as compared to all other groups. In addition, significant increases in the number of tumor-infiltrating immune cells, specifically cytotoxic T-cells, were observed in the TTFields plus anti-PD-1/anti-CTLA-4 or anti-PD-L1 groups. Correspondingly, cytotoxic T-cells isolated from these tumors showed higher levels of IFN-γ production. Collectively, these results suggest that TTFields have an immunoactivating role that may be leveraged for concomitant treatment with ICI to achieve better tumor control by enhancing antitumor immunity.

## 1. Introduction

The understanding of the basic interactions between the immune system and cancer has culminated in the development of new drugs over the past decade, most of which involve the blockade of immune checkpoint molecules [1]. These checkpoint molecules limit an over-extended immune response and allow tissue protection and maintenance of self-tolerance in normal tissues by physiologically decreasing the duration and amplitude of the immune response once it has been activated [1]. However, these molecules are often exploited by tumors as part of their immune evasion mechanisms. The inhibitory co-receptors cytotoxic T-lymphocyte-associated protein 4 (CTLA-4), programmed death-1 (PD-1), and the PD-1 ligands PD-L1 and PD-L2, serve as checkpoint pathways that mediate immunosuppression [2]. Drugs designed to block CTLA-4, PD-1, or the PD-1 ligands were shown to restore endogenous effector T-cell-mediated anti-tumor immunity [3]. The use of anti-CTLA-4, anti-PD-1, or anti-PD-L1 therapies facilitates T cell activation and prevents their exhaustion, thus facilitating an immune response against cancer [3,4].

An immunogenic tumor type, for which treatment with ICI is already FDA-approved, is non-small cell lung cancer (NSCLC) [5]. Approved ICI for treatment of unresectable stage III or metastatic NSCLC include several anti-PD-1 and anti-PD-L1 agents, and more recently, the combination of anti-PD-1 with anti-CTLA-4 has been added to this arsenal [6]. Possible differences between ICI agents could be clinically relevant for treatment tailoring based on tumor characteristics and acquired immune-related resistance [7]. While immunotherapy has drastically changed the landscape of NSCLC management, providing prolonged disease control, only a fraction of patients derive durable clinical benefits [8,9]. Whereas for patients with highly positive PD-L1 tumors (tumor proportion score, TPS ≥ 50%), single-agent immunotherapy has been consistently shown to be superior to chemotherapy, combination strategies are required for effectively treating patients with negative (<1%) or low-positive (1–49%) tumors, a population accounting for 70%–75% of NSCLC patients [10].

In order to sustain an effective anti-cancer response, the immune system needs to be activated in the first place. Combinations of immune checkpoint inhibitors (ICI) with agents that intervene at earlier steps of the cancer immunity cycle, addressing effective T-cell priming, removing barriers for T-cell trafficking and infiltration, or promoting tumor cell death in a way that is perceived as immunogenic, are beneficial [11]. Certain chemotherapeutic agents and modalities, such as radiotherapy and photodynamic therapy, were reported to induce immunogenic cell death (ICD), which results in the activation of dendritic cells (DCs) [12,13,14,15,16]. The activated DCs then migrate to the draining lymph nodes, present the cancerous antigens to T cells, and elicit adaptive immune responses [17]. Therefore, the induction of ICD facilitates the intervention of the immune system in growing tumors and drives into action the early steps of the cancer immunity cycle [18,19].

Tumor Treating Fields (TTFields) are low-intensity (1–3 V/cm), intermediate frequency (100–500 kHz) electric fields that exert physical forces to disrupt cellular processes critical for cancer cell replication and tumor progression [20,21,22]. TTFields therapy is FDA-approved and CE-marked for the treatment of adult patients with recurrent or newly diagnosed glioblastoma (GBM) and pleural mesothelioma. TTFields therapy is a locoregional, non-invasive cancer treatment delivered continuously using a portable electric field generator and insulated arrays that are applied to the skin around the tumor site with the intent to kill tumor cells and reduce local recurrence. In spite of the locoregional nature of this treatment modality, previous investigations have shown TTFields can exert an abscopal effect, suggesting induction of an immune-mediated anti-tumor response [23]. In-depth investigations have suggested the potential of TTFields to induce ICD in vitro and to initiate adaptive immunity by providing inflammatory stimuli for DCs [24,25]. As the anti-cancer immune response is a downstream effect of TTFields, this treatment modality may be a potential candidate for concomitant use with ICI. Indeed, TTFields concomitant with anti-PD-1 have shown efficacy in vivo [24] and are currently under clinical investigation for the treatment of NSCLC (LUNAR trial, NCT02973789; EF-36/KEYNOTE-B36 trial, NCT04892472).

In the current study, TTFields were shown for the first time to induce ICD in vivo and to be effective with concomitant anti-PD-1/anti-CTLA-4 or anti-PD-L1 therapy, supporting the emerging potential of TTFields therapy in the changing NSCLC immunotherapy landscape.

## 2. Results

### 2.1. TTFields Induce ICD In Vivo

**Figure 1:**
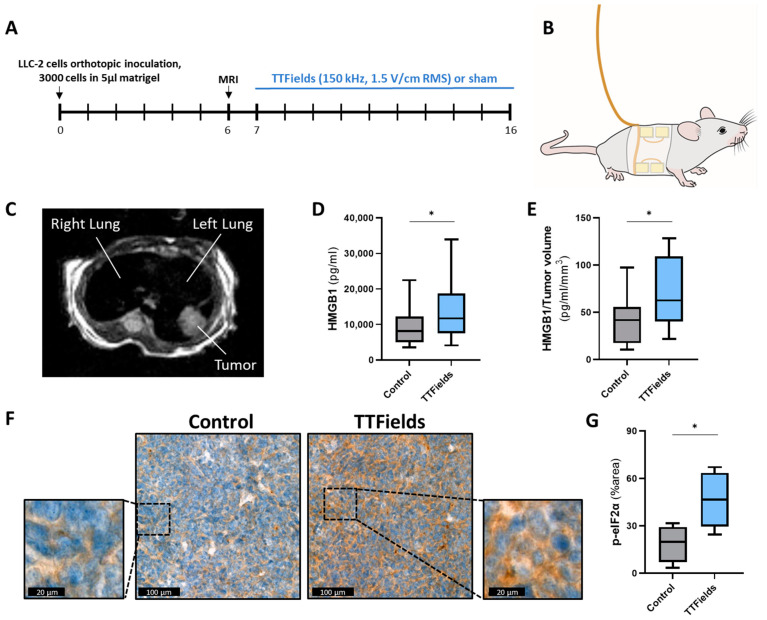
TTFields induced ICD in vivo. Mice were orthotopically inoculated with LLC-2 Lewis lung carcinoma cells, and TTFields or sham were delivered to the mice continuously for 10 days (**A**) via arrays placed symmetrically around their thorax (**B**). MRI was conducted at 6 days after tumor inoculation to verify the tumor was located within the lungs (**C**). At the end of treatment, circulating HMGB1 (**D**,**E**) and intratumoral p-eIF2α ((**F**,**G**) p-eIF2α in brown staining, and hematoxylin in blue staining) levels were measured. Values are mean ± SD. * *p <* 0.05; Student’s *t*-test.

To evaluate an immune response in vivo, immunologically compatible cancer cells must be inoculated into immunocompetent animals. The Lewis lung carcinoma (LLC)-2 model is a widely used syngeneic murine model for investigating lung cancer in animals, allowing for the examination of tumor growth and immune response [26]. To determine whether TTFields promote ICD in vivo, blood and tumor samples were collected from C57Bl/6 mice bearing orthotopic LLC-2 tumors treated for 10 days with sham or TTFields (Figure 1A–C and Figure S1). The samples were examined for ICD relevant biomarkers as follows: the alarmin high-mobility group box 1 (HMGB1), which is mandatory for efficient processing and cross-presentation of antigens from dying tumor cells [27,28,29]; and the phosphorylated form of the eukaryotic translation initiation factor 2α (p-eIF2α), which triggers calreticulin translocation to the cell surface of dying cells, where it serves as an engulfment signal for DCs [12,30,31,32]. Circulating levels of HMGB1 (Figure 1D,E) and intratumoral levels of p-eIF2α were significantly increased in TTFields-treated mice relative to control mice (Figure 1F,G), indicative of ICD induction by TTFields.

### 2.2. TTFields Concomitant with Anti-PD-1/Anti-CTLA-4 Were Effective In Vivo for Reducing Tumor Volume and Increasing Infiltration of Cytotoxic T Cells into the Tumor

**Figure 2:**
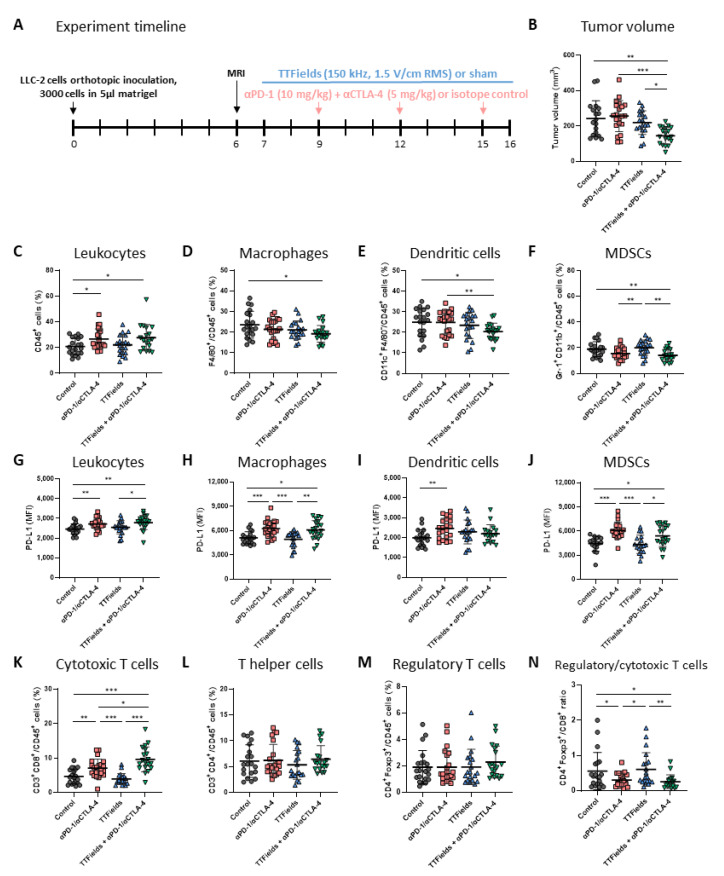
Concomitant TTFields with anti-PD-1/anti-CTLA-4 reduced tumor volume and increased tumor infiltration of cytotoxic T cells. (**A**) Mice were orthotopically inoculated with LLC-2 Lewis lung carcinoma cells and treated according to the depicted timeline with: (1) isotype control, sham; (2) anti-PD-1 + anti-CTLA-4, sham; (3) isotype control, TTFields; or (4) anti-PD-1 + anti-CTLA-4, TTFields. (**B**) Tumor volumes were measured at treatment end. Tumors were further analyzed for cell population abundance (**C**–**F**) and PD-L1 expression levels (**G**–**J**) of tumor leukocytes (CD45^+^) (**C**,**G**), macrophages (CD45^+^CD11b^+^F4/80^+^) (D,H), dendritic cells (CD45^+^CD11c^+^F4/80^–^) (**E**,**I**), and myeloid-derived suppressor cells (MDSCs, CD45^+^Gr-1^+^CD11b^+^) (**F**,**J**). The tumors were also analyzed for the percentages of cytotoxic (CD3^+^CD8^+^) (**K**), helper (CD3^+^CD4^+^) (**L**), and regulatory (CD4^+^Foxp3^+^) (**M**) T cells, and the ratio of regulatory to cytotoxic T cells was calculated (**N**). MFI-median fluorescence intensity. Three independent experiments are shown. n = 18–21 mice per group. Values are mean ± SD. * *p <* 0.05, ** *p <* 0.01, and *** *p <* 0.001; one-way ANOVA followed by Tukey’s multiple comparisons for tumor volume; Student’s *t*-test for flow cytometry immunophenotyping.

The effect of the concurrent application of TTFields and anti-PD-1/anti-CTLA-4 therapy was examined in C57Bl/6 mice orthotopically implanted with LLC-2 cells (Figure 2A). To allow the animals to heal from surgery before applying the arrays to their torsos and to better capture the stage at which patients are diagnosed in the clinic, treatment with TTFields was initiated only 7 days after inoculation; the first ICI injection was on day 9. The LLC-2 tumors displayed high aggressiveness, growing from about 10 mm^3^ on day 6 (as measured by MRI) to more than 200 mm^3^ in the control group 10 days later. Albeit these limitations, and although tumor volumes were similar in the control and anti-PD-1/anti-CTLA-4 alone groups and only slightly reduced following treatment with TTFields alone, tumor volume was significantly lower in the group treated with concurrent TTFields and anti-PD-1/anti-CTLA-4 relative to all other groups (Figure 2B).

Evaluation of immune cells in the mouse tumors showed that while treatment with TTFields alone did not elevate leukocyte (CD45^+^) infiltration, a significant increase was observed in both groups receiving anti-PD-1/anti-CTLA-4 injections, with or without concomitant TTFields (Figure 2C). Examination of the frequencies of specific myeloid populations in tumor tissue (Figure S2) surprisingly revealed significantly lower levels of macrophages (CD45^+^CD11b^+^F4/80^+^, Figure 2D) and DCs (CD45^+^CD11c^+^, Figure 2E) in tumors from the mice that were concomitantly treated with TTFields and anti-PD-1/anti-CTLA-4. Tumor levels of myeloid-derived suppressor cells (MDSCs, CD45^+^Gr-1^+^CD11b^+^, Figure 2F) were also significantly reduced in this group. These effects on myeloid cell infiltration were not seen in mice treated with TTFields alone or with anti-PD-1/anti-CTLA-4 alone.

In accordance with the elevated levels of leukocytes in the tumor and despite reductions in the levels of antigen-presenting cells (APCs) and MDSCs, PD-L1 expression levels were increased in leukocytes (Figure 2G), specifically macrophages (Figure 2H), DCs (Figure 2I), and MDSCs (Figure 2J) of tumors from mice treated with TTFields plus anti-PD-1/anti-CTLA-4 as compared to the control (*p*-value = 0.083 for DCs). Such PD-L1 elevations were also manifested at a similar level in the group treated with anti-PD-1/anti-CTLA-4 alone, whereas TTFields alone did not lead to a similar effect.

To directly address whether treatment with TTFields plus ICI promotes adaptive anti-tumor immunity, the presence of tumor-infiltrating lymphocytes (TILs) was examined (Figure S3). Whereas TTFields treatment alone did not affect the level of cytotoxic CD8^+^ T lymphocytes (CTLs) in the tumor, significant elevation was displayed in both groups treated with anti-PD-1/anti-CTLA-4 (Figure 2K and Figure S4A). Moreover, there was a significant increase in the level of CD8^+^ TILs in the group treated concomitantly with TTFields as compared with that for the group treated with anti-PD-1/anti-CTLA-4 alone. Levels of helper CD4^+^ and immunosuppressive regulatory CD4^+^Foxp3^+^ T cells (Figure 2L,M, respectively) were not significantly changed in any of the treatment groups, though signs of elevation were displayed by the group treated with TTFields plus anti-PD-1/anti-CTLA-4. As a result, the ratios of regulatory CD4^+^Foxp3^+^ to CD8^+^ TILs were significantly lower in the two groups treated with anti-PD-1/anti-CTLA-4 (Figure 2N).

### 2.3. TTFields Concomitant with Anti-PD-L1 Were Effective In Vivo for Reducing Tumor Volume and Increasing Infiltration of Cytotoxic T Cells into the Tumor

**Figure 3:**
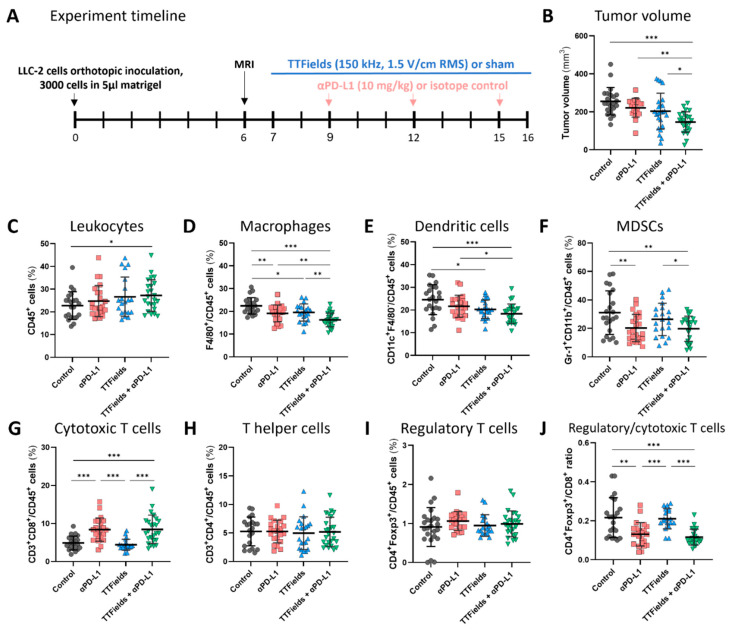
Concomitant TTFields with anti-PD-L1 reduced tumor volume and increased tumor infiltration of cytotoxic T cells. (**A**) Mice were orthotopically inoculated with LLC-2 Lewis lung carcinoma cells and treated according to the depicted timeline with: (1) isotype control, sham; (2) anti-PD-L1, sham; (3) isotype control, TTFields; or (4) anti-PD-L1, TTFields. (**B**) Tumor volumes were measured at treatment end. Tumors were further analyzed for the percentages of tumor leukocytes (CD45^+^) (**C**), macrophages (CD45^+^CD11b^+^F4/80^+^) (**D**), dendritic cells (CD45^+^CD11c^+^F4/80^–^) (**E**), myeloid-derived suppressor cells MDSCs (CD45^+^Gr-1^+^CD11b^+^) (**F**), as well as cytotoxic (CD3^+^CD8^+^) (**G**), helper (CD3^+^CD4^+^) (**H**), and regulatory (CD4^+^Foxp3^+^) (**I**) T cells, and the ratio of regulatory to cytotoxic T cells was calculated (**J**). MFI-median fluorescence intensity. Three independent experiments are shown. n = 14–17 mice per group. Values are mean ± SD. * *p <* 0.05, ** *p <* 0.01, and *** *p <* 0.001; one-way ANOVA followed by Tukey’s multiple comparisons for tumor volume; Student’s *t*-test for flow cytometry immunophenotyping.

Similar results were obtained when anti-PD-L1 was employed together with TTFields in C57Bl/6 mice orthotopically implanted with LLC-2 cells (Figure 3A). Mice treated with monotherapies (anti-PD-L1 or TTFields) demonstrated a mild decrease in tumor volume as compared to the control group, while concomitant treatment of TTFields and anti-PD-L1 led to a significant decrease in tumor volume as compared to the other 3 groups (Figure 3B). The mice treated with anti-PD-L1 alone did not demonstrate a significant elevation of leukocyte (CD45^+^) infiltration into the tumor relative to the control, whereas a significant increase was observed in the group receiving TTFields with concomitant anti-PD-L1 (Figure 3C). In addition, tumors from the mice that were concomitantly treated with TTFields and anti-PD-L1 displayed significantly lower levels of macrophages (Figure 3D), DCs (Figure 3E), and MDSCs (Figure 3F) relative to the control, and in the case of macrophages and DCs, also relative to anti-PD-L1 monotherapy. Increased tumor leukocytes with reduced levels of macrophages, DCs, and MDSC were also seen in the groups treated with the monotherapies, but to a lesser extent. PD-L1 levels could not be examined in this study since this receptor was blocked by the treatment antibody. Whereas no changes in tumor CTL levels were seen in the group treated with TTFields alone, significant elevation was displayed in both groups treated with anti-PD-L1 (Figure 3G and Figrue S4B). Unlike what was observed with TTFields plus anti-PD-1/anti-CTLA-4, TTFields did not significantly enhance anti-PD-L1-induced CTL infiltration. No changes were seen in the levels of T helper TILs in any of the treatment groups (Figure 3H), as well as in the levels of regulatory CD4^+^Foxp3^+^ cells (Figure 3I), whereas the CD4^+^Foxp3^+^ to CD8^+^ ratio was significantly lower in the two groups treated with anti-PD-L1 (Figure 3J).

### 2.4. Concurrent TTFields and ICI Increased the Effector Function of Tumor-Infiltrating T Cells

**Figure 4:**
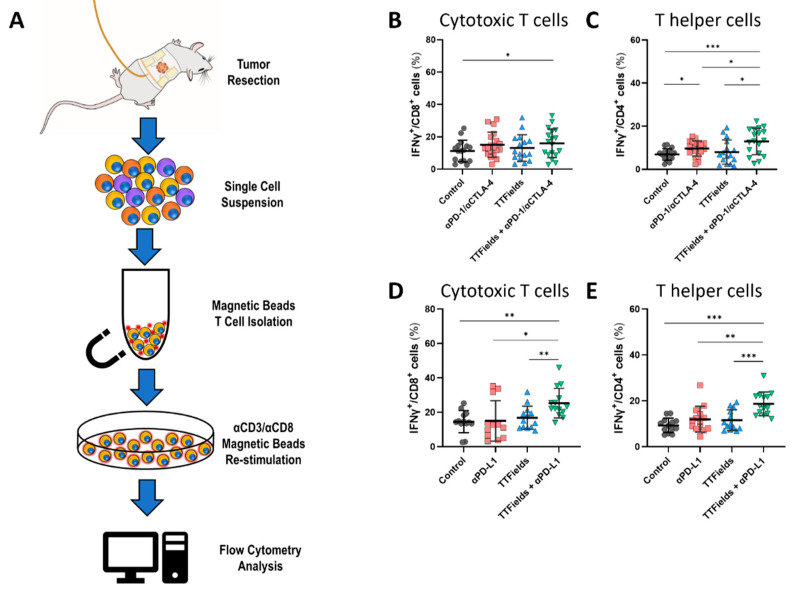
Co-application of TTFields and ICI increased the effector function of tumor-infiltrating T cells. (**A**) Tumor-infiltrating lymphocytes (TILs) were isolated and stimulated, and intracellular IFN-γ levels were examined. IFN-γ levels produced by CD8^+^ (**B**) and CD4^+^ (**C**) TILs from the anti-PD-1/anti CTLA-4 (αPD-1/αCTLA-4) experimental set. IFN-γ levels produced by CD8^+^ (**D**) and CD4^+^ (**E**) TILs from the anti-PD-L1 (αPD-L1) experimental set. Values are mean ± SD. * *p <* 0.05, ** *p <* 0.01, and *** *p <* 0.001; Student’s *t*-test.

TILs isolated from tumor tissue were activated using CD3/CD28 and stained for T cell markers and IFN-γ (Figure 4A and Figure S5). Significant elevation relative to the control of IFN-γ production by stimulated CD8^+^ TILs was seen in the group treated concurrently with TTFields and anti-PD-1/anti-CTLA-4, but not in the groups treated with TTFields or anti-PD-1/anti-CTLA-4 alone (Figure 4B). IFN-γ production by CD4^+^ TILs derived from the TTFields plus anti-PD-1/anti-CTLA-4 treatment group was significantly elevated relative to all other study groups (Figure 4C). Significant elevation in IFN-γ production by stimulated CD8^+^ and CD4^+^ TILs (Figure 4D,E, respectively) was also seen in the group treated with TTFields and anti-PD-L1 relative to all other treatment groups.

### 2.5. TTFields Application Did Not Impede ICI-Derived Formation of Splenic and Blood Effector Memory Cytotoxic T Cells

**Figure 5:**
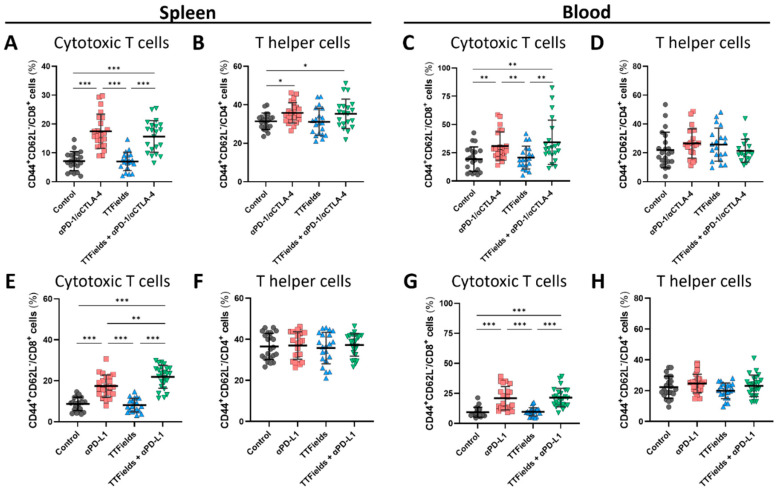
TTFields application did not impede ICI-derived formation of splenic and blood effector memory cytotoxic T cells. Spleen (**A**,**B**,**E**,**F**) and blood (**C**,**D**,**G**,**H**) were collected from the anti-PD-1/anti CTLA-4 (αPD-1/αCTLA-4) experimental set mice (**A**–**D**) and from the anti-PD-L1 (αPD-L1) experimental set mice (**E**–**H**), and analyzed for the percentages of effector memory cytotoxic (CD3^+^CD8^+^) (**A**,**C**,**E**,**G**) and helper (CD3^+^CD4^+^) (**B**,**D**,**F**,**H**) T cells. Values are mean ± SD. * *p <* 0.05, ** *p <* 0.01, and *** *p <* 0.001; Student’s *t*-test.

To assess the formation of peripheral immune memory following treatment with TTFields plus ICI (Figure S6), blood and spleen were examined, yielding a somewhat similar pattern to that seen for the TILs. Specifically, levels of effector CTLs (CD8^+^CD44^+^CD62L^–^) and effector T helper cells (CD4^+^CD44^+^CD62L^–^) in the spleen were not affected by TTFields alone but were significantly elevated in both groups treated with anti-PD-1/anti-CTLA-4 (Figure 5A,B, respectively). An increase in effector CTLs (Figure 5C) was seen in the blood of animals from both groups treated with anti-PD-1/anti-CTLA-4, which was somewhat higher in the TTFields-conjunct group, while no significant effects were seen in the level of effector T helper cells in any treatment group (Figure 5D).

Levels of splenic effector CTLs increased following anti-PD-L1 treatment relative to control. This elevation was significantly higher with TTFields plus anti-PD-L1 than with anti-PD-L1 alone (Figure 5E). However, anti-PD-L1 treatment did not have any effect on the level of effector T helper cells in the spleen (Figure 5F). Effector CTL levels were also elevated in the blood of animals from the two groups treated with anti-PD-L1 (Figure 5G), while there were no changes in the level of effector T helper cells in the blood (Figure 5H).

## 3. Discussion

The current research aimed to examine in vivo the potential of TTFields, a treatment modality that has shown immunostimulatory effects, with concomitant ICI for the treatment of NSCLC. The first step was to confirm that the ability of TTFields to induce ICD, observed in vitro [24], is also manifested in vivo (Figure 1). A surrogate biomarker of ICD is the release of HMGB1 from dying tumor cells, which is required for the processing and cross-presentation of antigens [27,28,29] and for the recruitment of T cells to the tumor microenvironment (TME) [33,34]. In the current study, levels of HMGB1 in the blood were significantly elevated when TTFields were applied, suggesting the release of HMGB1 by dying tumor cells. This is the first report demonstrating elevated HMGB1, not only locally, as previously demonstrated in cell supernatants, but also at the systemic level. While in certain types of cancer, high levels of circulating HMGB1 have been associated with a poorer prognosis [35], the elevation of HMGB1 following treatment was previously demonstrated to correlate with improved treatment outcomes [36]. Another central event in the course of ICD induction is the activation of the endoplasmic reticulum (ER) stress response. In particular, eIF2α phosphorylation is required for cell surface exposure of the ER chaperone calreticulin, which facilitates the transfer of tumor-associated antigens to DCs [12,30,31,32]. Assessing the degree of phosphorylation of eIF2α provides a convenient parameter to monitor ICD. In the current study, in vivo TTFields application resulted in the phosphorylation of eIF2α. Overall, the results demonstrate for the first time that TTFields contribute to cancer immunosurveillance in vivo.

Next, the efficacy of the concomitant application of TTFields and ICI was tested (Figure 2 and Figure 3). Overall, treatment with TTFields, anti-PD-1/anti-CTLA-4, or anti-PD-L1 alone provided limited effects on tumor growth, in accordance with the reported poor immunogenicity of this model [37]. Despite the high aggressiveness displayed by the tumors, the relatively delayed treatment initiation, and the limited effect of the individual therapies, the co-application of TTFields with either ICI treatment strategy significantly reduced tumor growth as compared to control as well as to each treatment alone.

The concomitant treatment with TTFields and ICI resulted in changes in the TME (Figure 2 and Figure 3). An elevation in leukocyte infiltration into the tumor due to an increase in the level of CTLs was seen. On the other hand, the levels of macrophages and MDSCs, as well as the CD8^+^ to regulatory TILs ratio, were reduced in tumors from animals treated with anti-PD-1/anti-CTLA-4 or anti-PD-L1, and more so when TTFields were co-applied with either ICI strategy. While in vitro investigations examining the direct application of TTFields to T cells have demonstrated some level of proliferation inhibition of CD8^+^ and CD4^+^ cells without affecting any of their other anti-tumoral functions [38], in the current in vivo study, TTFields did not seem to impede tumor T cell levels. This is in line with previous observations, showing that while TTFields induce an anti-migratory effect on tumor cells, they do not impair random or chemoattractant-induced leukocyte migration [39]. In the clinical setting, high tumor infiltration of CD8^+^ lymphocytes following treatment with anti-PD-1 therapy was reported and was found to be associated with favorable overall and progression-free survival [40,41]. On the other hand, the accumulation of macrophages, MDSCs, and regulatory T cells in the TME, which potentially mediates immunosuppressive activity in the tumor, was associated with worse disease outcomes [42,43,44]. Overall, the augmentation of CTL levels together with the attenuation of macrophage and MDSC levels in the TEM support the improved tumor control in the TTFields-ICI concomitant treatment groups.

In the anti-PD-1/anti-CTLA-4 experiment, although myeloid cell infiltration was reduced, PD-L1 expression by these cells, and overall in the tumor, was elevated (Figure 2). While PD-L1 expression is a pivotal escape mechanism of tumor cells from the immune system, an increase in PD-L1 following treatment suggests an adaptive immune attempt to limit the inflammatory response [45]. Indeed, clinical response to PD-L1 and PD-1 blockade has been associated with increased PD-L1 expression in the tumor tissues [41,46]. More specifically, PD-L1 expression on APCs in the TEM, particularly macrophages and DCs, rather than cancer cell-intrinsic PD-L1, has been suggested to account for the potential therapeutic efficacy of PD-1/PD-L1 signaling blockade [46]. Overall, the results presented in this work support the notion that TTFields applied concomitantly with ICI elicit an adaptive immune attempt to limit the inflammatory response.

The most profound effect seen in the current study was higher levels of ex vivo IFN-γ production by the CD8^+^ and CD4^+^ TILs in both experimental sets (Figure 4). Manifestation of this property in vivo suggests increased IFN-γ levels in the tumor milieu, supporting the aforementioned elevation in PD-L1 density in leukocytes from tumors in the concomitant treatment groups [41,47]. Though no changes were seen in the level of T helper cells in the tumor, the elevation in IFN-γ production by the CD4^+^ cells suggests a tilt in the balance between the anti-tumorigenic type 1 and the immunosuppressive type 2 helper cells in the TME, in favor of the former. The proinflammatory capacity of peripheral lymphocytes, mainly their IFN-γ production, was suggested to be a predictive marker for inhibition of tumor progression and preferable survival outcomes following PD-1 or dual PD-1/CTLA-4 blockade [40,41,47]. Taken together, these results suggest that the addition of TTFields concomitantly with ICI boosts the immunoactivity of the TME.

Another important observation of the current research was the elevation of effector memory CTLs in the spleen and blood (Figure 5). A previous investigation in GBM cells suggested that pro-inflammatory cytokines are released by cells treated with TTFields, promoting the short-term formation of CD8^+^ effector T cells in mice inoculated with the TTFields-treated cells, and long-term elevation in central memory T cells (CD44^+^CD62L^+^) [25]. While the short duration of the study presented here does not allow the formation of central memory [48], the results do suggest the potential for immune memory to be formed after TTFields application. The elevation of effector memory T cells in the blood and spleen, together with the increase in TILs, may suggest a treatment-evoked specific anti-tumor immune response.

A previous study reporting on the effects of TTFields with concomitant anti-PD-1 in the LLC mouse model revealed elevated levels of tumor leukocytes, which were attributed mainly to an increase in the number of macrophages and DCs [24]. However, in the current study, tumor levels of macrophages and DCs were reduced, while levels of TILs were elevated and accompanied by the formation of effector memory T cells. These differences may be ascribed to the longer treatment duration used in the current study, which allowed the detection of more long-term effects. Supporting this claim are results from a longer examination of TTFields and anti-PD-1 in a CT-26 colon cancer model, in which a significant reduction was seen in the level of tumor DCs with a significant elevation in the levels of TILs [24].

While current and past in vivo studies could not demonstrate changes in the immune milieu of tumors treated with TTFields monotherapy, results from GBM patients treated with TTFields concomitant with chemotherapy suggest a shift from a pro- to an anti-tumor immune signature [25,38]. Those studies compared tumor tissue samples and peripheral blood mononuclear cells (PBMCs) from patients with newly diagnosed GBM before and after treatment with TTFields and concomitant standard temozolomide chemotherapy. The studies demonstrated increased CD8^+^ infiltration into the tumors, upregulation of DCs and innate immune cells, IFN-γ-induced activation of DCs, peripheral T cell clonal expansion, and CD8^+^ T cell activation towards memory development. Overall, those studies demonstrated effective adaptive immunity activation in GBM patients treated with TTFields.

Despite recent success in many cancer types, immunotherapeutic approaches continue to encounter challenges in “cold” tumors such as GBM, mainly due to the highly immunosuppressive environment of such tumors [5,49,50]. Nonetheless, preliminary clinical data from the 2-THE-TOP phase 2 pilot clinical study, in which TTFields therapy was applied to patients with newly diagnosed GBM together with temozolomide and anti-PD-1 therapy, demonstrated improved efficacy, as well as robust post-TTFields adaptive immunity activation [51]. The efficacy of TTFields therapy concomitant with ICI for the treatment of patients with NSCLC is currently under investigation, in the LUNAR (NCT02973789) and EF-36/KEYNOTE-B36 (NCT04892472) trials.

In conclusion, TTFields promote ICD of tumor cells, and when delivered concomitantly with ICI may augment the immune response, via higher tumor infiltration, IFN-γ production, and development of effector memory CD8^+^ T cells, promoting anti-tumor immunity, and resulting in improved tumor control.

## 4. Materials and Methods

### 4.1. Tumor Cell Lines

Murine Lewis lung carcinoma cell line LLC-2 was obtained from the American Type Culture Collection (ATCC).

### 4.2. Design of Animal Experiments

Animal housing and anesthesia as well as array composition and placement procedure were previously described (Figure S1A,B) [23]. In brief, 10–12-week-old male C57Bl/6 mice (Harlan Laboratories, Jerusalem, Israel) were injected directly into the lungs with LLC-2 cells (3 × 10^3^/5 µL) suspended in high concentration growth factor reduced Matrigel (1:1 volume; BD Biosciences, Bedford, MA, USA). Tumor volume and location were examined by magnetic resonance imaging (MRI) 6 days after inoculation, and only mice with verified localized tumors were included in the experiments (Figure 1C). Animals were randomized into the following four study groups: (1) isotype control, sham; (2) ICI, sham; (3) isotype control, TTFields; or (4) ICI, TTFields. In one set of experiments, mice received intraperitoneal (I.P.) injections of anti-PD-1 (Bio X cell, Lebanon, NH, USA; RPM1-14; 10 mg/kg) with anti-CTL4 (Bio X cell, Lebanon, NH, USA; 9H10; 5 mg/kg), or Rat Ig2a (Bio X cell, Lebanon, NH, USA; 2A3; 10 mg/kg) with polyclonal Syrian Hamster IgG (Bio X cell Lebanon, NH, USA; BP0087; 5 mg/kg) for isotope control on days 9, 12, and 15 from inoculation. In the second set of experiments the same timeline was employed, with I.P. injections of anti-PD-L1 (Bio X cell, Lebanon, NH, USA; 10F.9G2; 10 mg/kg) or Rat IgG2b (Bio X cell, Lebanon, NH, USA; LTF-2; 10 mg/kg) for isotope control. In both settings TTFields (1.86 ± 0.67 V/cm RMS [52]; 150 kHz; inovivo system, Novocure, Haifa, Israel) were applied continuously for 10 days starting at day 7 from tumor inoculation [20,23]. Device usage data were recorded to ensure animals successfully received therapy for ≥ 18 h/day, as per clinical recommendations for maximizing treatment benefits [24,25]. Sham arrays were identical in size and shape to the TTFields arrays, generated equivalent heat (38.5 °C), and were placed on the thorax of the animals at the same orientation as the treatment arrays.

### 4.3. MRI Set Up

MRI was acquired using the ICON 1 Tesla MRI system (Bruker Biospin, Ettlingen, Germany). Mice were anesthetized with isoflurane and placed in a dedicated body coil in a prone position. Transverse T2 weighted RARE images were acquired with the following parameters: repetition time—2000 ms; excitation time—56 ms; RARE factor—12; number of scans—8; number of slices—12; slices thickness—0.9 mm; matrix size—128 × 128; FOV—28 mm; in-plane resolution—0.22 × 0.22 mm; acquisition time—3 min 12 s.

### 4.4. Tissue Processing

At the end of the treatment, mice were anesthetized and blood was obtained by submandibular bleeding. Whole blood was incubated with red blood cells BD Pharm Lyse™ (BD Biosciences, Bedford, MA, USA) lysis buffer and washed. For serum, blood was collected in tubes without anticoagulant and kept at room temperature for up to 30 min for clotting. The tubes were then spun in a refrigerated centrifuge (4 °C) at 2000× *g* and the supernatant collected. Then, the mice were euthanized, and spleens and tumors were dissected. Spleens were dispersed through a 70 μm nylon cell strainer, incubated with red blood cells lysis buffer (BD Pharm Lyse™), and washed. Tumors were dissected, measured, and either submerged in OCT (Fisher Scientific, Hampton, NH, USA; Scigen 4586), formalin-fixed and paraffin-embedded, or prepared as single-cell suspensions using the gentleMACS^TM^ dissociator and the tumor dissociation kit, as per manufacturer’s instructions (Miltenyi Biotec, Bergisch Gladbach, Germany), and dispersed through a 70 μM nylon cell strainer.

### 4.5. Detection of HMGB1

HMGB1 was quantified by ELISA assay on the sera from control and TTFields-treated mice, according to the manufacturer’s instructions (IBL International GmbH).

### 4.6. Immunohistochemistry

Immunohistochemical (IHC) staining was performed on fresh-frozen and OCT-embedded 15-µm-thick tumor sections using the Leica BOND-MAX system (Leica Biosystems Ltd., Newcastle, UK). Tissues were pretreated with BOND epitope-retrieval solution for 20 min, followed by 30 min incubation with anti-CD8 (SinoBiological, Beijing, China; 50389-R309) primary antibody or anti-phospho-eIF2α (S51) antibody (Cell Signaling, Danvers, MA, USA; 3398S). The Leica Refine-HRP kit (Leica Biosystems, Newcastle, UK; DS9800) was used for detection and counter-staining with hematoxylin. Slides were scanned, and non-tumor areas excluded using the CaseViewer software. The signals of the stained protein and the nuclei were resolved by color deconvolution and quantified separately using ImageJ software. Average signal per cell or percent of positive cells was calculated.

### 4.7. Measurements of Tumor Volume

Tumors were measured with Vernier calipers, and their volume calculated using the formula width^2^ × length × 0.5.

### 4.8. Flow Cytometry

Viobility 405/452 Fixable Dye (Miltenyi Biotec, Bergisch Gladbach, Germany) was used for the discrimination of dead cells. Mouse Fc block (Biolegend, San Diego, CA, USA; anti-CD16/CD32 (clone 93)) was used prior to staining with the fluorochrome-conjugated anti-mouse antibodies listed in Table 1. For staining of receptors and/or intracellular lineage-defining factors, Cyto-Fast™ Fix/Perm Buffer Set (Biolegend, San Diego, CA, USA) was utilized according to manufacturer instructions. Intracellular FOXP3 staining was performed using Foxp3 Fix/Perm buffer kit (Biolegend, San Diego, CA, USA) or FoxP3 Staining Buffer Set (Miltenyi Biotec, Bergisch Gladbach, Germany) following the manufacturer protocol. Data were acquired on MACSQuant Analyzer 10 (Miltenyi Biotec, Bergisch Gladbach, Germany) flow cytometer and analyzed using FlowJo software (BD Biosciences, Bedford, MA, USA). The gating strategy is outlined in Figures S2, S3, S5 and S6.

### 4.9. Isolation and Activation of Tumor-Infiltrating Leukocytes (TILs)

TILs were isolated from filtered tumor tissue single-cell suspensions by treatment with Mouse Pan T (CD90.2) magnetic bead selection (Invitrogen, Waltham, MA, USA). Isolated cells were then cultured in vitro in the presence of T-Activator CD3/CD28 magnetic beads (Invitrogen, Waltham, MA, USA) for 24 h. Brefeldin A (Biolegend, San Diego, CA, USA) was added to the culture 4 h before staining with antibodies against CD8, CD4, and CD3, followed by permeabilization with Cyto-Fast™ Fix/Perm Buffer Set (Biolegend, San Diego, CA, USA) and staining with antibodies against IFNγ.

### 4.10. Statistical Analysis

Data are presented as mean ± standard deviation (SD). Statistical significance was calculated using GraphPad Prism 8 software (La Jolla, San Diego, CA, USA), with the specific tests used mentioned in figure legends. Differences were considered to be statistically significant for *p* values of ≤0.05 and were indicated as * *p* <  0.05; ** *p*  <  0.01; *** *p*  <  0.001.

## Figures and Tables

**Table 1:** Staining reagents.

Antigen	Fluorochrome	Cat #	Manufacturer
CD11b	PerCP	101320	Biolegend
CD11c	PE/Cy7	117318	Biolegend
CD16/32	NA	101320	Biolegend
CD3	PE/Cy7	100220	Biolegend
CD4	APC/Fire 750	100460	Biolegend
CD44	APC	103012	Biolegend
CD45	VioGreen	130-110-665	Miltenyi Biotec
CD62L	PE/Cy7	104418	Biolegend
CD8	FITC	100723	Biolegend
F4/80	APC/Fire 750	123152	Biolegend
F4/80	PE	123110	Biolegend
FoxP3	PE	320008	Biolegend
GR1	Alexa Fluor 647	108418	Biolegend
IFNγ	Alexa 647	505810	Biolegend
PD-L1	PE	124308	Biolegend
Viobility	405/452	130-130-403	Miltenyi Biotec

## Data Availability

All data generated or analyzed during this study are included in this article. Further inquiries can be directed to the corresponding authors.

## Supplementary Materials

The supporting information can be downloaded at: https://www.mdpi.com/article/10.3390/ijms232214073/s1.
