# Supplementary material for: Tumor Treating Fields (TTFields) Concomitant with Immune Checkpoint Inhibitors Are Therapeutically Effective in Non-Small Cell Lung Cancer (NSCLC) In Vivo Model
Source: Int J Mol Sci. 2022 Nov 15;23(22):14073. doi: 10.3390/ijms232214073 (PMC9696536; doi:10.3390/ijms232214073)
Supplement: Supplementary file 1 [file ijms-23-14073-s001.zip › ijms-1980646-supplementary.pdf]

Figure S1

A

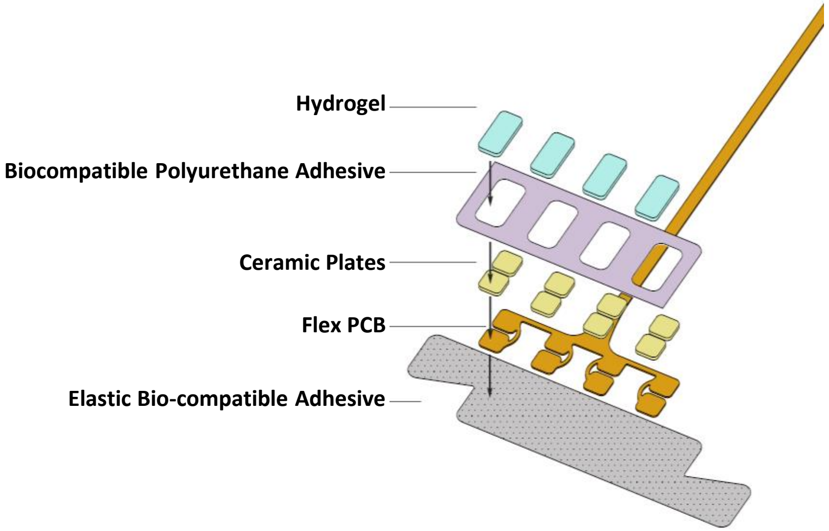

B

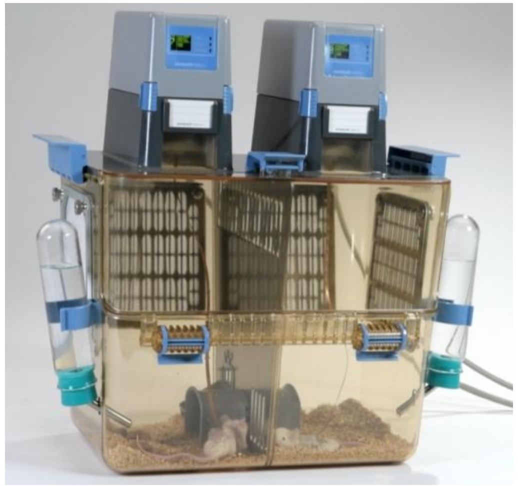

(A) Electrode array design. Each array consisted of 2 ceramic disks, and the 4 arrays were compacted into one printed circuit board (PCB) cable. **(B)** Image of a cage used in the study. Cages were designed to house 2 mice separated by bars to prevent cable entanglement, but allow dyadic interactions of the mice.

Figure S2

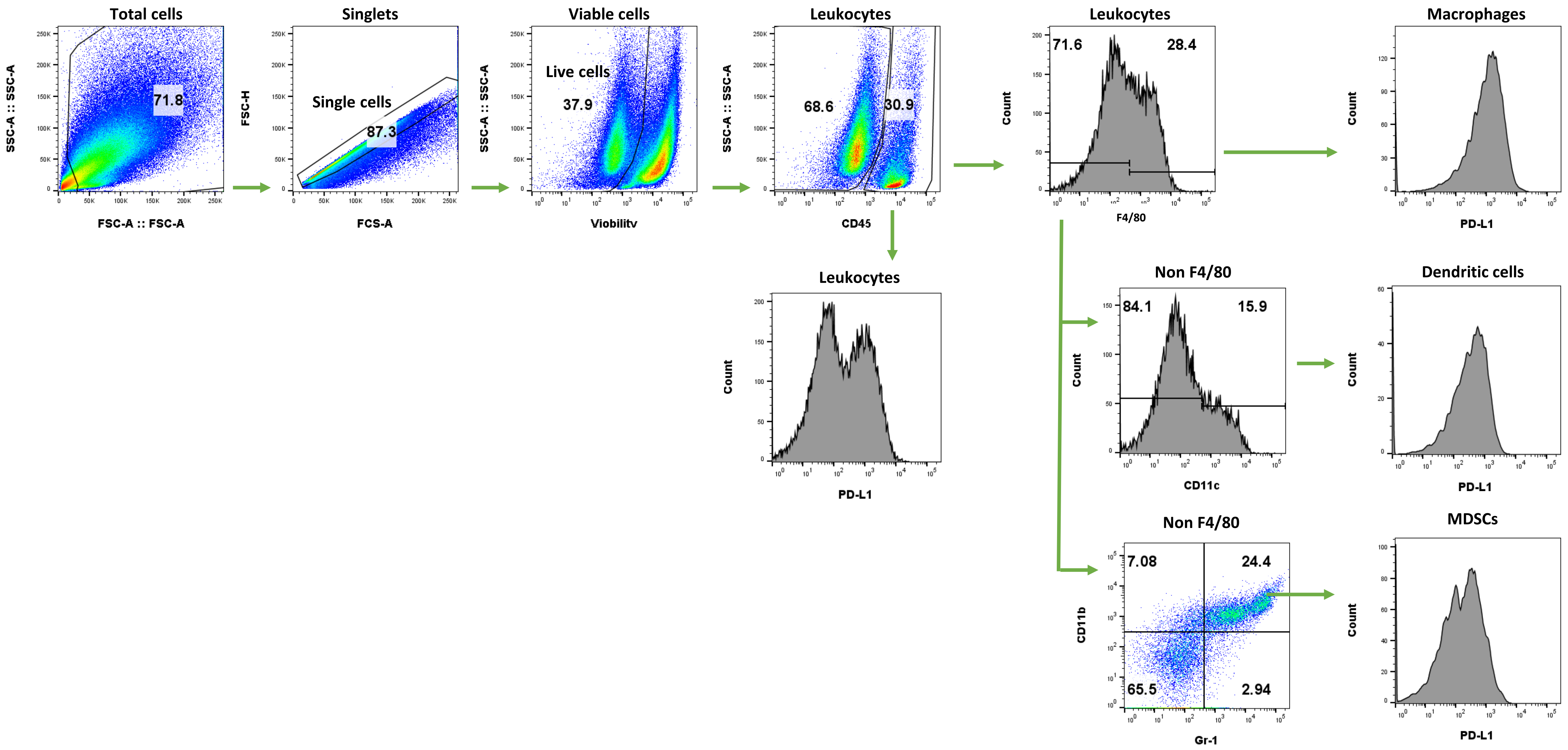

Flow cytometric gating strategy for discerning innate subsets. Leukocytes were gated based on CD45 expression. $CD45^+$ cells were further divided into macrophages ($CD45^+F4/80^+CD11b^+$), dendritic cells ($CD45^+CD11c^+$), and myeloid-derived suppressor cells (MDSCs, $CD45^+Gr\text{-}1^+CD11b^+$) subsets. All populations were further gated on the basis of PD-L1 expression.

Figure S3

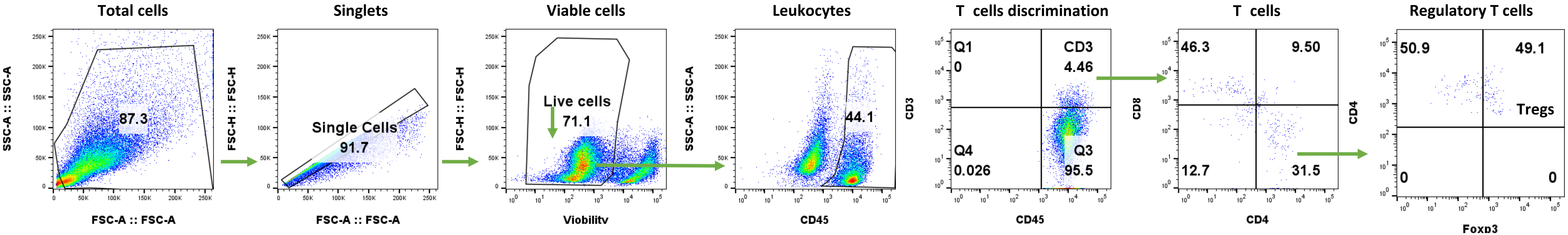

Flow cytometric gating strategy for discerning T cell subsets. T cells were gated based on CD45 and CD3 expression, and then further divided into cytotoxic T cells ($CD45^+CD3^+CD8^+$), helper T cells ($CD45^+CD3^+CD4^+$), and regulatory T cells ($CD45^+CD3^+CD4^+Foxp3^+$).

Figure S4

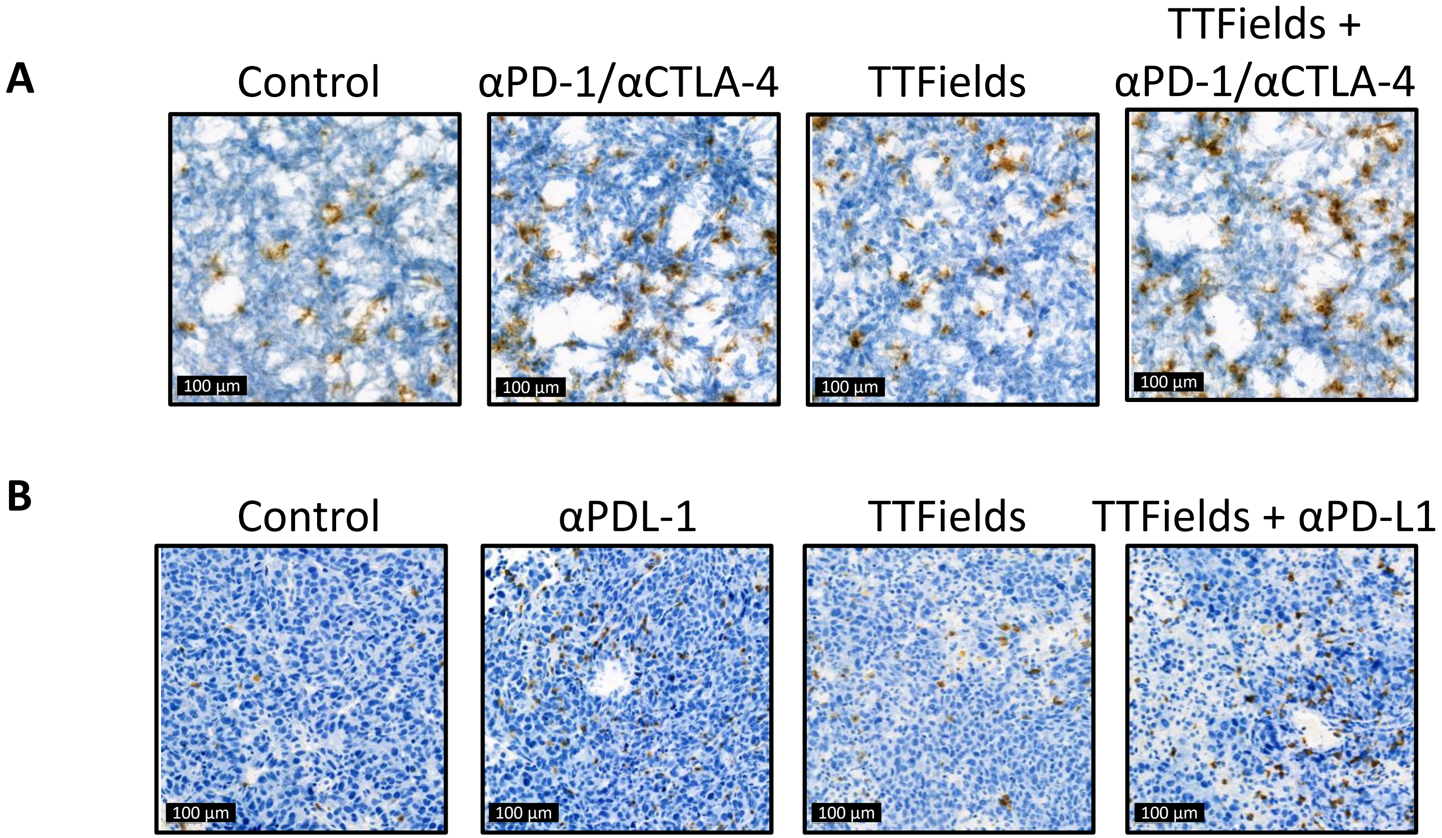

Immunohistochemical staining for $CD8^+$ cells (brown staining) in fresh-frozen tumor sections of mice from the anti-PD-1/anti-CTLA-4 (αPD-1/αCTLA-4) experiment **(A)** and in formalin-fixed tumor sections of mice from the anti-PD-L1 (αPD-L1) experiment **(B)**. Counter-staining with hematoxylin (blue staining) was used for cell detection.

Figure S5

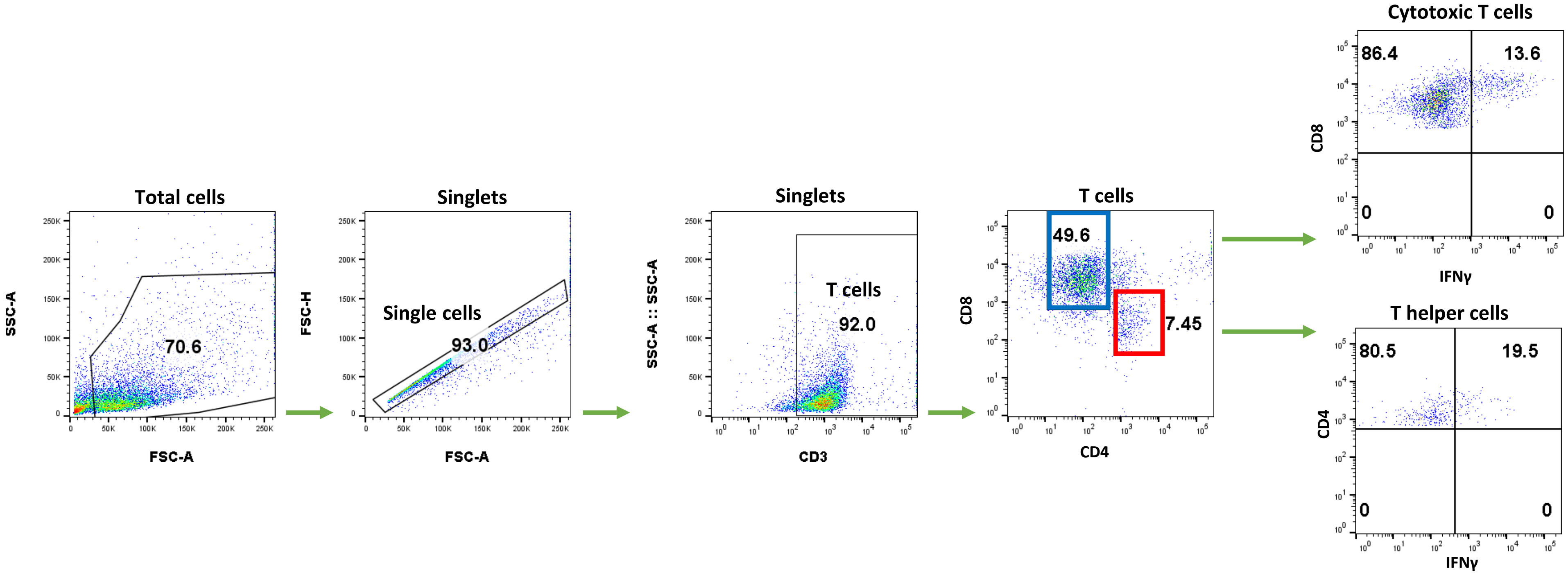

Flow cytometric gating strategy for detecting T cell IFN-γ production. T cells were gated based on CD3 expression. $CD3^+$ cells were further divided into cytotoxic ($CD3^+CD8^+$) and helper ($CD3^+CD4^+$) sets, from which IFN-γ level were determined.

Figure S6

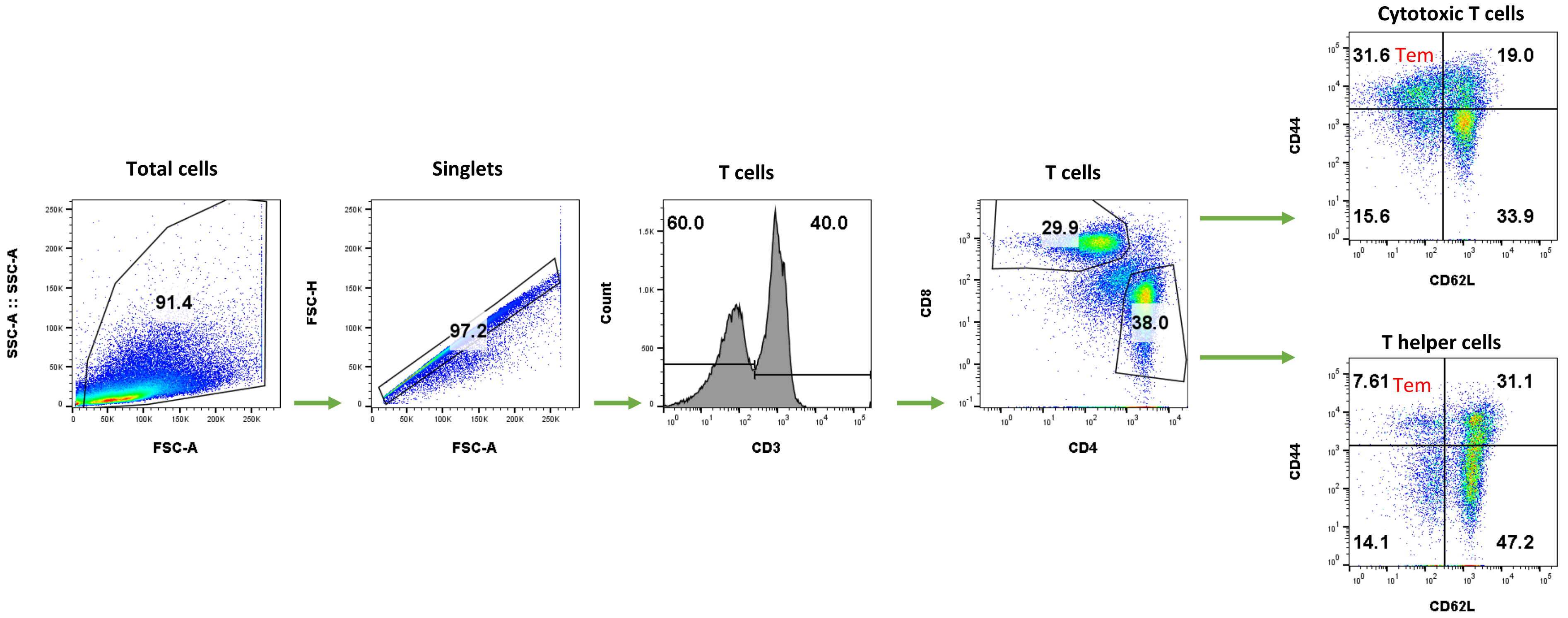

Flow cytometric gating strategy for discerning effector memory T cell subsets. T cells were gated based on CD3 expression. $CD3^+$ cells were further divided into cytotoxic ($CD3^+CD8^+$) and helper ($CD3^+CD4^+$) sets, from which effector memory cells were identified ($CD44^+CD62L^-$).
